# Twenty-fifth Annual Report of the Bloomingdale Asylum for the Insane

**Published:** 1846-04

**Authors:** 


1. *Twenty-fifth Annual Report of the Bloomingdale Asylum for the Insane.* By PLINY EARLE, M. D., Physician to the Asylum. New York. 1846. pp. 48.

2. *Report of the Pennsylvania Hospital for the Insane, for the year* 1845. By THOMAS S. KIRKBRIDE, M. D., Physician to the Institution. Philadelphia. 1846. pp. 56.

3. *Thirteenth Annual Report of the Trustees of the State (Massachusetts) Lunatic Hospital at Worcester, December,* 1845. Boston. 1846. pp. 106.

4. *Third Annual Report of the Managers of the State (N.Y.) Lunatic Asylum, made to the Legislature January* 23, 1846. Albany, 1846. pp. 61.

The Reports emanating from the Institutions for the Insane, which have been provided in some of the States, are well adapted to diffusing information not only amongst physicians, but amongst the general community. That the number of persons attacked with insanity has considerably increased within a few years seems manifest, and that it has done so in a ratio exceeding that of the increase of population, there are reasons for believing. These institutions have been instrumental in restoring to health and usefulness thousands of persons, whose lives, without their aid, would have been a burden to their friends or to the community; but in this, perhaps, they have not rendered a greater service than they are capable of doing, by giving, through their annual reports, more correct views than are generally entertained of the nature of insanity, of its treatment; and by extending our knowledge of its predisposing and exciting causes, a proper understanding of which may do much towards diminishing the prevalence of this distressing malady.

1. The following table from Dr. Earle's Report, gives the general statistics of the *Bloomingdale Asylum* for the past year.

| | | Males. | Females. | Total. |
|---|---|---|---|---|
| Number of patients in the Asylum Jan. 1, 1845, | | 54 | 50 | 104 |
| Number of cases admitted during the year, | | 71 | 67 | 138 |
| Whole number in the Asylum during the year, | | 125 | 117 | 242 |
| Number of cases discharged and died, | | 65 | 60 | 125 |
| Remaining, December 31st, 1845, | | 60 | 57 | 117 |
| Of the cases discharged there were cured, | | 30 | 31 | 61 |
| " " " " | Much improved, | 3 | 9 | 12 |
| " " " " | Improved, | 17 | 3 | 20 |
| " " " " | Unimproved, | 8 | 12 | 20 |
| " " " " | Died, | 7 | 5 | 12 |

Table V gives a comparative view of the exciting causes of the malady, physical and moral. According to it there are fifty-nine physical, of which intemperance, self-abuse amongst the men, and the circumstances attending the puerperal state amongst the females, are the principal: and thirty-six moral, in which pecuniary losses, disappointments, &c., and religious and political excitement predominate.

In making this comparison the report says, "it is possible that a large proportion of the cases arranged under the head of physical causes in the above table, might be traced to some agent acting upon the mind. Intemperance, cerebral disease, epilepsy, typhus fever, may be induced, and frequently are, by mental influences. With this view of the subject, the relative numbers in the two classes of causes might be essentially varied."

In speaking of the increased prevalence of insanity, as shown by the increase of patients in the Asylum, the Dr. says: "Whether the increase be in a greater ratio than that of the population of the city and its adjacent country, is a proposition which cannot be easily demonstrated. However this may be, it is an unquestionable fact, that the exciting causes of mental alienation were never in time of peace more active among any people than at the present day among the inhabitants of the United States, and particularly so in the States which, bordering on the Atlantic, were the earliest peopled by European emigrants.

"Intoxicating liquors are so cheap, that the labor of a few hours will procure enough to addle the brain for a week, and prevent the healthy exercise of reason perhaps a much longer period. The avenues to wealth, place and power, are open to all: the child of the cottager, thus entering into the strife of competition with the son of the most wealthy citizen. The progress of civilization and refinement, and the comparative ease with which the products of both nature and art in every quarter of the globe are here obtained, have a direct tendency to foster a luxurious life. Hence, human desires and human wants are greatly multiplied, while both body and mind are exerted to the utmost power of endurance to gratify the former and supply the latter. The almost unavoidable effect of the artificial mode of living thus produced, is either a debility of the system, or an augmentation of nervous excitability, either of which facilitates the invasion of mental disease.

"Art has made advances so rapid towards the annihilation of time and space, that, if life be measured by the proper standard, the number of events, circumstances and conditions, seen, felt and perceived—the amount of pleasure enjoyed and pain endured—the people of the present generation have an existence of ten-fold longer duration than their forefathers. As if this were not enough, the mind is forced into an activity corresponding with the new era of art. Children, before the body has acquired sufficient tone, or the brain sufficient firmness, to endure much mental exertion with impunity, are placed in schools where the intellectual faculties are unduly urged; while the physical exercise necessary to the due development of the frame is too often neglected. Under such circumstances the head will expand, but the body cannot grow in size or vigor sufficiently to maintain 'a balance of power.'

"If the child escape the more immediate dangers thus produced, he arrives at manhood with an unnatural susceptibility of mental excitement, as well as increased disposition to diseases of the brain, by what causes soever they may be induced."

Of the one hundred and nineteen cases admitted during the year, "the disease in sixty-eight commenced within one year previous to the time of admission, while in fifty one it had existed more than a year. The most important practical fact connected with the duration of the disease, is its influence upon the curability of the patient. Of the recent cases admitted, thirty-two have been discharged cured; and of those that remain, nineteen are believed to be curable. Of the chronic cases, only seven have been discharged cured, and of the remainder only eight are considered curable. These facts alone exhibit the importance of treatment in the early stages of mental disorder."

There are other matters of interest contained in the report which might claim attention. Amongst these are the various means of moral treatment made use of, such as religious worship, the school lectures, amusements, manual labor, &c., but the limits of a notice like this compel us to pass them by.

2. From the Report of the *Pennsylvania Hospital*, we learn that there were 151 patients in the Institution at the date of the previous report, and that 177 have been admitted, and 159 have been discharged or died since that time, leaving 169 under care at the close of the year. The highest number at one time was 174; the average number for the whole year was 162, being more than at any previous period in the history of the Institution.

"Of those discharged during the year 1845, were—

| | |
|---|---|
| Cured, - - - - | 80 |
| Much improved, - - | 5 |
| Improved, - - - - - | 24 |
| Stationary, - - - | 30 |
| Died, - - - - | 20 |
| Total, - | 159 |

"Of the patients discharged cured, thirty-nine were residents of the Hospital not exceeding three months; twenty-six between three and six months; twelve between six months and one year; and three for a longer period than one year."

A part of the report is occupied with the statistics of 769 patients who have entered the Institution, showing the age at which insanity first appeared, the cause, form, duration of the disease, &c., but as they are not sufficient in themselves to afford any correct general results, we pass them over.

Table XIII shows the state of 600 patients who have been discharged or died,—their sex, and the form of disease for which they were admitted.

| | Males. | Females. | Total. | Mania. | Melancholia. | Monomania. | Dementia. | Delirium. |
|---|---|---|---|---|---|---|---|---|
| Cured, | 188 | 125 | 313 | 189 | 62 | 57 | 5 | -- |
| Much improved, | 28 | 22 | 50 | 28 | 9 | 10 | 3 | -- |
| Improved, | 51 | 28 | 79 | 36 | 20 | 12 | 11 | -- |
| Stationary, | 51 | 37 | 88 | 32 | 19 | 13 | 23 | 1 |
| Died, | 43 | 27 | 70 | 31 | 17 | 2 | 15 | 5 |

"By 'cured' is meant, that the patient's mind has been restored to what was its natural state before the accession of the disease, which brought him to the Hospital. Natural eccentricities of character do not constitute insanity, and these, of course, are not expected to be removed by a residence in any Institution. The friends of patients have occasionally believed their minds to be in a better state after recovery from an attack of insanity, than they were before its accession; and there are highly respectable professional men who have spoken of the same results. This, however, is hardly to be anticipated in any case; but where the recovery has been considered complete, after a certain period, the mind has in most cases appeared to me to have all the strength and integrity that originally belonged to it."

In relation to a cause of death formerly more common amongst the insane than at present, the Dr. observes:

"Consumption was formerly one of the most common terminations of life with old cases of insanity, and was supposed by many to be a consequence of mental disease. It is much more probable, however, that it was the result of the treatment too often adopted. Close confinement in badly ventilated rooms, want of exercise in the open air, and sometimes a meagre diet, would certainly tend to develope those diseases where there existed the slightest constitutional tendency to them. We would naturally expect what has been here fully realized, the most striking changes in the physical condition of this whole class, by having them constantly out of their chambers, except at night, spending several hours of each day in walking or working in the open fields, and at all times with a wholesome nutritious diet.

"My observations during the past five years, induce me to believe that a proper system of discipline and regimen will make the occurrence of tubercular and other diseases of a kindred character, quite as rare, perhaps more rare in insane hospitals, than with the mass of the community."

The remainder of the report is principally devoted to a consideration of the circumstances and modes of treatment capable of exerting a beneficial moral influence on patients in an institution of the kind. "The principle has always been recognised in this Institution, that it is of great importance that the grounds about a hospital should be well improved, and in such a state of cultivation as to attract the interest of the patients, and each year, as less rough work has been required on the premises, greater attention has been paid to this species of improvement. Much useful labour has thus been afforded to a considerable number, and occupation of a light kind to many who are not among the votaries of labour. All take some degree of interest in these operations. Extended walks, enlarged flower borders, handsome summer houses, and improvements about the water courses, are among the objects that will be permanently of interest to our patients." "A succession of improvements about the buildings and grounds, keeps up a steady interest in those who work; and to those who do not, proof that efforts are constantly being made to promote their comfort and happiness."

"The plan for a regular course of lectures, referred to in my last report, has since been fully matured, and is now being carried out with the most gratifying success." "As far as it can be conveniently effected, it is intended these lectures shall be demonstrative, and rendered attractive by as great a variety of illustrations as can be appropriately introduced. It has been found, however, that these adjuvants are not always necessary; and the attention of the class has been well kept up, even where a lecture of fifty minutes has been mostly made up of rather dry details, or somewhat abstruse philosophical definitions."

The reports of Dr. Kirkbride are always eminently practical, embodying the results of his experience and observation in whatever tends to promote the interests of his patients and the insane generally. Our space will not permit us to make further extracts; we must, therefore, refer to the report itself, for other various matters which we are obliged to omit.

3. The *Massachusetts Hospital*, at Worcester, has been enlarged since last year, by the addition of apartments for one hundred and sixty patients, and now possesses accommodations for nearly four hundred, the benefits of which are enjoyed by three hundred and sixty insane—a larger number than are collected together in any similar institution in this country. The report for the past year, like its predecessors, contains a large amount of valuable statistical and other information, from the pen of its Superintendant, Dr. Woodward.

| | Men. | Women. | Total. |
|---|---|---|---|
| Number of patients in the house in the course of the year, | 292 | 264 | 556 |
| " at the commencement of the year, | 128 | 135 | 263 |
| " admitted in the course of the year, | 164 | 129 | 293 |
| " remaining at the end of the year, | 192 | 168 | 360 |
| " discharged during the year, | 100 | 96 | 196 |
| " recent cases discharged cured, | 57 | 47 | 104 |
| " of whom were cured, | 49 | 44 | 93 |
| " chronic cases discharged, | 43 | 49 | 92 |
| " of whom were cured, | 15 | 14 | 29 |

Table X shows the causes of insanity, and circumstances connected with causes and predisposition to insanity, and is as follows:

| | |
|---|---|
| Intemperance, | 278 |
| Ill health, | 318 |
| Masturbation, | 145 |
| Domestic afflictions, | 219 |
| Religion, | 191 |
| Property, | 131 |
| Disappointed affections, | 68 |
| Disappointed ambition, | 33 |
| Epilepsy, | 56 |
| Puerperal, | 67 |
| Wounds of the head, | 26 |
| Abuse of snuff and tobacco, | 9 |
| Jealousy, | 11 |
| Fright, | 14 |
| Palsy, | 19 |
| Hereditary, or having insane kindred or ancestors, | 565 |
| Periodical, | 450 |
| Homicidal, | 23 |
| Have committed homicide, | 16 |
| Suicidal, | 239 |
| Have committed suicide, | 11 |
| Arising from physical causes, | 918 |
| From moral causes, | 667 |

Many not classed.

The following remarks are of some practical importance.

"The condition of the brain is rendered such by the intemperate use of alcoholic drinks, that even a suspension of their use will not always prevent the occurrence of insanity. With great opportunities for observation, I do not now recollect a single case of delirium tremens arising from the abandonment of intoxicating drinks. I know that many preventives have been prescribed, and that a cautious withdrawal has been recommended to prevent this calamity—yet in my experience of six years as physician of a prison, and thirteen as Superintendant of this Hospital, I have seen many individuals who were broken off abruptly from all stimulating drinks, yet I do not think a single case of this disease has occurred. The reformation of intemperate persons by the modern temperance reform, sustains this view of the subject, so contrary to former theories of this disease. The work of disease is so far advanced in some individuals who have recently reformed, or have pretended to reform from the use of intoxicating drinks, that ordinary insanity has occurred without the apparent intervention of any other causes,—even in those cases the withdrawal of the stimulus cannot be reasonably supposed to have had any agency in producing the insanity."

Dr. Woodward says that, in his experience, the senses, and especially the sense of hearing, have been affected with illusions, in cases of insanity arising from intemperance, and relates several instances of this kind. He then continues:

"Alcohol is not the only narcotic which thus affects the brain and nervous system. Opium produces delirium tremens, and probably insanity. *Tobacco* is a powerful narcotic agent, and its use is very deleterious to the nervous system, producing tremors, vertigo, faintness, palpitation of the heart, and other serious diseases. That tobacco certainly produces insanity I am not able positively to observe; but that it produces a predisposition to it I am fully confident. Its influence upon the brain and nervous system generally, is hardly less obvious than that of alcohol, and if excessively used, is equally injurious. The young are particularly susceptible to the influence of these narcotics. If a young man becomes intemperate before he is twenty years of age, he rarely lives to thirty. If a young man uses tobacco while the system is greatly susceptible to its influence, he will not be likely to escape injurious effects, that will be developed sooner or later, and both diminish the enjoyments of life and shorten its period."

"In our experience in this Hospital, tobacco, in all its forms, is injurious to the insane. It increases excitement of the nervous system in many cases, deranges the stomach, and produces vertigo, tremors, and stupor in others."

Table XII shows the diseases which have proved fatal in 175 cases.

| | |
|---|---|
| Marasmus, - - - - - - | 37 |
| Apoplexy and Palsy, - - - - - | 20 |
| Epilepsy, - - - - - - | 17 |
| Consumption, - - - - - - | 16 |
| Disease of the heart, - - - - - | 13 |
| Suicide, - - - - - - - | 11 |
| Disease of the brain, - - - - | 7 |
| Typhus fever, - - - - - | 6 |
| Hemorrhage, - - - - - - | 5 |
| Lung fever, - - - - - - | 5 |
| Cholera morbus, - - - - - | 4 |
| Inflammation of bowels, - - - - | 4 |
| Dysenteric fever, - - - - - | 4 |
| Mortification of limbs, - - - - | 3 |
| Dropsy, - - - - - - - | 3 |
| Diarrhœa, - - - - - - - | 3 |
| Chronic dysentery, - - - - - | 3 |
| Erysipelas, - - - - - - | 3 |
| Disease of the brain from intemperance, - | 2 |
| Bronchitis, - - - - - - | 2 |
| Old age, - - - - - - - | 1 |
| Gastric fever, - - - - - - | 1 |
| Land scurvy, - - - - - - | 1 |
| Congestive fever, - - - - - | 1 |
| Concussion of the brain, - - - - | 1 |
| Disease of the bladder, - - - - | 1 |
| Fright, - - - - - - - | 1 |
| Total, - - - - - | 175 |

The above table is interesting as showing the fatal affections of the insane, to which their disease renders them liable.

Table XVIII shows the causes of insanity as affecting persons pursuing different occupations, and affords some curious results, for which we must refer to the report itself. Then follow some remarks on the effects of education and proper training in preventing insanity.

"When there exists a strong constitutional tendency to insanity, it is a question of serious magnitude to decide what course to pursue with offspring to ward off the danger, and protect the system from its accession. All that can be said of hereditary predisposition is, that it increases the liability to disease; it is never, strictly speaking, a cause. Some other influence must be brought to bear upon the individual before he will have gout, consumption, scrofula, epilepsy, insanity, or any other hereditary disease. The course adopted by parents and teachers in this matter is often a mistaken one. Strong propensities should generally be checked, not encouraged. The student should be taught to be active, and the active be restrained and made to study. If the nervous system is exceedingly susceptible, the muscular and circulating systems should be encouraged to activity, so as to balance this unequal tendency. If the child is disposed to be rough, passionate, and quarrelsome, he should be subjected to gentle influences, mild diet, and female society and instruction. Obedience to well directed authority is essential to any suitable control and solitary management. Unrestrained passion, ungoverned appetite, and unlimited indulgence, are probably more frequently the causes of insanity, diseases of the heart, and other severe nervous affections, than hereditary predisposition; they establish a predisposition where none existed, and may also prove an exciting cause of disease in the very cases in which they have given the tendency.

"There are many cases of children where the mind is too active for the body; intense application wears upon the physical powers and induces disease. In others, all the energies of the system are devoted to gratify animal wants and the lower propensities. Of all things, precocity should be discouraged; the very indication of it is a proof of incipient disease of the brain, which, if encouraged, will inevitably result in concentrating the elements of death upon the brain or some vital organ. The common impression that children must be permitted to pursue their own inclinations in the business of life is not always sound discretion. The character should be moulded by training, and the inclinations be changed when they tend to develope the causes of disease which hereditary propensity or acquired tendency has established. This can always be done by suitable training, and this is the great business of education. It is not so much to store the mind with knowledge, as to train the faculties, lop off excrescences, and cultivate what is feeble and improperly developed, thus making a well balanced mind, fortified to meet the evils of life, to overcome difficulties, and to bear with good spirit the unavoidable calamities that will meet us. Knowledge can be acquired at any age, but the foundation of a useful and happy life must be laid in youth, when the susceptibilities are tender, and the confidence in parents and teachers, leading to no suspicions of error, is permanent and abiding. Many of the evils of life, the imperfections of character, the sufferings to which after-life is incident, and many of the failures of youth to accomplish anticipated results, arise from education, founded on incorrect principles, and pursued to the accomplishment of undesirable ends. Such are also the foundations of disease, and especially insanity. When we witness the great number of cases of insanity arising from improper influences, education, habits, unrestrained desires and propensities, we have little cause to seek for natural tendencies, but great reason to lament over evils now established in the character and life, which an early, correct discipline would have averted."

Dr. Woodward has been making a series of observations to ascertain the influence the state of the moon may have in exciting paroxysms in cases of periodical insanity. He gives the results of his experience in 125 cases of this form of the disease, in which he has observed 875 paroxysms. Of 86 new paroxysms of excitement, which were noted during the year, 51 occurred at the new and full moon, and 35 at the quadratures,—a result, as the Dr. says, more favourable to the theories which have been promulgated on the subject of lunar influence, but as a whole not sufficient to sustain them. As this is a subject at the present time of close philosophical investigation, he considers that the inquiry should be encouraged, rather than ridiculed and rejected.

4. From the Report of Dr. Brigham we learn, that the number of patients in the *Asylum at Utica*, at the beginning of the year, was

| | Men. | Women. | Total. |
|---|---|---|---|
| | 131 | 129 | 260 |
| Admitted during the year, | 151 | 142 | 293 |
| Total number in the course of the year, | 282 | 271 | 553 |
| Discharged during the year, | 139 | 129 | 268 |
| Of whom were recovered, | 76 | 59 | 135 |
| " improved, | 39 | 39 | 78 |
| " unimproved, | 14 | 20 | 34 |
| " died, | 10 | 11 | 21 |
| Remaining at the end of the year, | 143 | 142 | 285 |

Of 844 patients received since the opening of the Asylum, 409 were married, 392 single, 31 widows, and 12 widowers. The report says: "We continue to receive more married than single persons, while most Asylums in the country receive more single than married. We do not know the cause of this difference."

The following extracts on the subject of mental occupation are worthy of notice.

"Our observation for many years, in various lunatic Asylums, led us a long time since to regard the want of *mental occupation* as the greatest want in modern institutions for the insane. Go into any such establishment, and you will find some few, in winter a very few, at work—some playing cards or other games; yet a still larger class will be found sitting about, listless, inactive, doing nothing, saying nothing, taking no interest in anything going on around them, gathering around the stove, or place that is heated, looking forward to nothing but the hour of eating and retiring to sleep. For a short time each day, when the physician passes around, they will exhibit a little animation, and say a few words, and then relapse into their former condition.

"When the weather is pleasant, some of them walk or ride out occasionally for a short time, but this, to many of the class we are describing, after a few times, seems to be a mechanical kind of business, and confers but little enjoyment; they notice nothing, and say nothing, during the walk or ride, or after it. These patients make but little trouble in an Asylum, and are very apt to be overlooked and neglected, and if not already demented, soon become so. They are thought not to require much attention, as they have good bodily health, and are quiet, consequently they generally receive but little notice.

"*But this class require great attention;* they need mental exercise; they should attend school, and have their minds aroused into mental activity for an hour or two every day. Soon, by this course, their memories will improve; they will become interested in singing or study, and by perseverance soon will be cured, and many, very many, rendered capable of much enjoyment, and be kept from sinking into a state of hopeless dementia.

"Governed by such views, we have rarely repressed any new method proposed by the patients themselves for exercising and improving their minds. Hence we have a *debating society* that meets once a week, which is conducted not only with good order, but with ability. Occasionally original plays are acted. Albums are circulated, also a weekly newspaper, handsomely printed with a pen; all of which interest and amuse many, and are harm to none.

"In addition to maps and a globe, geographies and historical works for the school, when those that are about will attend, we have a large library, from which the patients obtain books three times in the week. We have also a very large supply of newspapers and magazines.

In some of the halls reading parties are formed, for the purpose of reading aloud new and useful works; as, for instance, at the present time, in our hall, the "Narrative of the United States Exploring Expedition" is being read; and in another, Wiley & Putnam's Library of Choice Reading."

"By these means we have the satisfaction of seeing many patients not only recover from their mental disorder, but that their minds have been improved, a fact of which they themselves are conscious, and for which they are grateful. In repeated instances we have been informed, by the relatives and neighbors of patients who have here recovered and gone home, of their increased intelligence and marked improvement of mind."

Dr. Brigham considers that "we should not remain satisfied with providing curative institutions and comfortable abodes for those affected by insanity, we should endeavour to prevent it by timely care and wise precautions, by avoiding whatever is likely to predispose to it, or to excite it." With the hope that some suggestions on the subject may be useful, he devotes a portion of his report to some remarks on the "prevention of insanity." In this connexion he deems it proper to allude to *the neglect of the study of insanity by Physicians.* We give the following extracts:

"Many physicians, in general practice, are, we think, too apt to consider insanity a disease with which they have little or nothing to do. Hence they often neglect to qualify themselves by study and careful observation to treat it properly. Their attention is not directed to it, the same as to other diseases, by lectures in the medical schools, and they seldom purchase and read the best treatises on the subject.

"It is very true that, in most cases, when the disease becomes established, it cannot be well treated at home. Patients often become prejudiced against their nearest friends, and will not hearken to their advice nor take any medicine. For these reasons, and to remove them from the exciting causes of their disease, as well as to have them treated properly in all respects, it is usually necessary to place them in an asylum. Still there are instances, though they are not common, where none of these objections nor any other exist to the patient remaining at home, and being treated by his ordinary physician. It is, therefore, very necessary that physicians in general practice should qualify themselves to prescribe in cases of insanity, as well as in other diseases.

"But, admitting that no such cases occur, it is still important for them to thus prepare themselves in order to prevent insanity, and to *arrest* it in its incipient stage.

"We have no hesitation in saying, that if the physicians of the country were fully aware of their duty to the community in this respect, and would exert themselves to prevent insanity by timely advice, and to arrest it in its early stage, that they would do to those predisposed to insanity, and to the insane themselves, an amount of good unequalled by that of the asylums of the country.

"They should understand, and be able to recognize, its earliest symptoms; for, as has been said, insanity often, and we believe we may say most generally, exists in a slight and scarcely perceptible degree for months before it is generally noticed. They should know how liable many are to this disease from hereditary predisposition, from previous attacks, long continued menorrhagia or other diseases, from repelled eruptions and extreme nervous susceptibility, and be able to advise such, and warn them in time of impending danger. How many cases of puerperal insanity, or of that insanity that comes on after child-birth, might be prevented by timely precautions?

"Physicians should study the various causes of mental diseases, and learn the danger of over-excitement of the nervous system, especially in early life, by too strong emotions, by prematurely tasking the intellectual powers, by the improper indulgence of the appetites and passions, and by the neglect of moral and religious education; and thus be able to advise parents and others whenever they are pursuing a course likely to lead to this disease.

"In this way physicians would do great good in individual cases, and also very much towards arresting those alarming *epidemic delusions* that occasionally prevail through the country; a lamentable instance of which is within the knowledge of all, known by the name of 'Millerism,' or the doctrine of the immediate destruction of the world. This has not only sent many to the grave, and rendered a vast number insane, but predisposed, we apprehend, a large number, by the excitement and terror it has produced, to nervous diseases, and to insanity hereafter.

"Physicians are often called upon to give their testimony in relation to the mental condition of individuals, and sometimes in cases where not only property but *life* is at stake. In such cases their responsibility is very great, and furnishes strong additional reasons for their applying themselves diligently to the study of mental diseases."

We cannot, we think, do better than to conclude these notices with the words of Dr. Brigham. "These brief and imperfect (?) views of the causes of insanity and means of prevention, we hope will not be valueless to the community. They certainly will not, if they awaken the attention of others to the same subjects—subjects well worthy the most careful attention of the patriot and philanthropist." J. H. W.

